# Importance of Marine-Derived Nutrients Supplied by Planktivorous Seabirds to High Arctic Tundra Plant Communities

**DOI:** 10.1371/journal.pone.0154950

**Published:** 2016-05-05

**Authors:** Adrian Zwolicki, Katarzyna Zmudczyńska-Skarbek, Pierre Richard, Lech Stempniewicz

**Affiliations:** 1 Dept. of Vertebrate Ecology and Zoology, University of Gdańsk, Wita Stwosza 59, 80–308, Gdańsk, Poland; 2 Littoral, Environnement et Sociétés, UMR 7266 CNRS – Université de La Rochelle, 2 rue Olympe de Gouges, 17000, La Rochelle, France; Estación Biológica de Doñana, CSIC, SPAIN

## Abstract

We studied the relative importance of several environmental factors for tundra plant communities in five locations across Svalbard (High Arctic) that differed in geographical location, oceanographic and climatic influence, and soil characteristics. The amount of marine-derived nitrogen in the soil supplied by seabirds was locally the most important of the studied environmental factors influencing the tundra plant community. We found a strong positive correlation between *δ*^15^N isotopic values and total N content in the soil, confirming the fundamental role of marine-derived matter to the generally nutrient-poor Arctic tundra ecosystem. We also recorded a strong correlation between the *δ*^15^N values of soil and of the tissues of vascular plants and mosses, but not of lichens. The relationship between soil *δ*^15^N values and vascular plant cover was linear. In the case of mosses, the percentage ground cover reached maximum around a soil *δ* ^15^N value of 8‰, as did plant community diversity. This soil *δ*^15^N value clearly separated the occurrence of plants with low nitrogen tolerance (e.g. *Salix polaris*) from those predominating on high N content soils (e.g. *Cerastium arcticum*, *Poa alpina*). Large colonies of planktivorous little auks have a great influence on Arctic tundra vegetation, either through enhancing plant abundance or in shaping plant community composition at a local scale.

## Introduction

Polar terrestrial ecosystems are distinctive, as they develop and function under very harsh conditions [1]. Among the main recognized ecological factors affecting the development and dynamics of terrestrial vegetation in polar regions are: air temperature [2,3], soil moisture [4,5], soil pH [5], nutrient availability [6], snow cover [7], proglacial chronosequences [8], dispersal limitations [9] and natural disturbances [4]. These factors are modulated at the macroclimatic scale, e.g. resulting from geographical separation, as well as by microclimatic features such as topography (elevation and exposure) [10] and, in turn, influence the structure and distribution of polar vegetation [11,12].

The tundra ecosystem is generally poor in nutrients [1]. However, at a local scale, marine birds and mammals deposit large amounts of excrement (rich in nitrogen, phosphorus and other elements) near their breeding colonies or permanent resting sites, fertilizing these areas [13–15]. Nutrients delivered in this way play an important role in defining local soil chemical characteristics, enhancing vegetation productivity and biomass, and influencing the spatial distribution of plants [13–17]. Ornithogenic compounds assimilated by plants are subsequently transferred to higher trophic levels and finally, through decomposition, back to soil [18,19].

Little auks (dovekies, *Alle alle*) play a potentially crucial role in Arctic tundra development. They are the most numerous and widespread High Arctic seabird, with a global population of ca. 37 million pairs [20]. The Svalbard population alone has been estimated to comprise more than 1 million pairs [20]. Their role as biovectors is that they forage in the sea and deposit droppings on land. The food of little auks is comprised mostly of copepods (*Calanus glacialis* and *C*. *hyperboreus* in cold Arctic waters, and *C*. *finmarchicus* in warmer Atlantic waters) [21]. During the breeding season, little auks supply up to ca. 60 t km^-2^ dry mass of faecal matter in the vicinity of their colony in Hornsund (south-west Spitsbergen) [22]. Our previous study [15] showed that such large levels of nutrient deposition have strong influence on soil physical and chemical parameters there, and also create a steep fertility gradient from the colony to the sea.

It is well known [23,24] that nitrogen and carbon play fundamental roles in the growth and development of plants, and are amongst the most important resources in biogeochemical cycles. Depending on whether these elements are of marine or terrestrial origin, the ratios of their stable isotopes (^15^N/^14^N expressed as *δ*^15^N, and ^13^C/^12^C as *δ*^13^C, respectively) are different [23]. Since the proportion of heavier isotopes is generally greater in marine organic matter, and further increases when passing through successive trophic levels, in this study we used *δ*^15^N and *δ*^13^C signatures of soil and plant tissues as indicators to identify seabird influence [25,26].

Although the effects of seabird colonies on tundra vegetation have been widely reported [16,18,27,28], there are no quantitative studies comparing the combined response (competitive ability) of specific plant species, or the entire tundra plant community, to multiple environmental factors affecting them at the same time. In this study we tested the relative importance of the following factors for tundra development: (1) total nitrogen and carbon content and isotopic signatures of soil, (2) soil characteristics (moisture, pH, conductivity), and (3) geographical location.

We test the hypothesis that nutrients derived from the marine ecosystem via colonial seabirds are locally the main factor determining the abundance and structure of plant communities in Svalbard. The other aims of this study were also to identify plant species most dependent on birds’ nutrient enrichment and to describe their response along the fertilization gradient.

## Materials and Methods

### Ethical statement

The study was performed under Governor of Svalbard permission nos. 2007/00150-2 a.512, 2007/00150-5 and 2007/00150-9. Tissue collection was undertaken on non-endangered plant species.

### Study area

**Fig 1:**
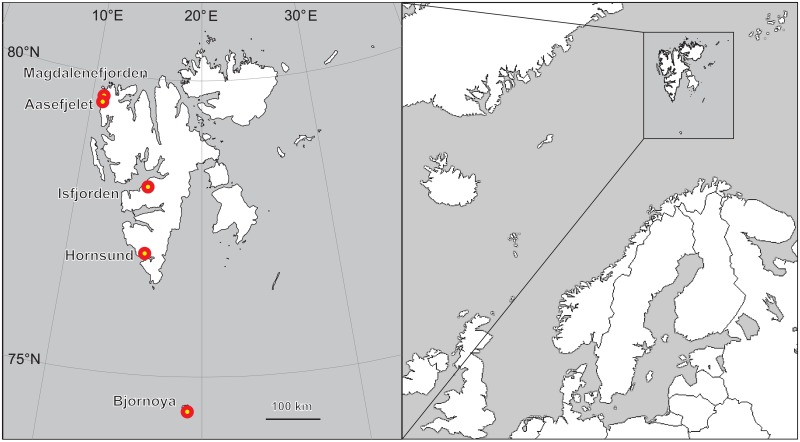
Study area locations within the Svalbard archipelago.

The study was conducted at five locations within the Svalbard archipelago, differing in local climate regimes and the sizes of local little auk populations (Table 1, Fig 1).

**Table 1:** Description of five study locations.

Study location	LatLon	Colony size (pairs)*	No of transects	Transect description	Climatic conditions
Magdalenefjorden	79.58°N 11.03°E	18000	3	Høystakken—south-west, descending to sea inside the fjord, 9 plots; Høystakken—west, descending to glacial moraine, 7 plots; Skarpegga—south-west, descending to sea inside the fjord, 8 plots.	Influenced by warm Atlantic water masses carried by the West Spitsbergen Current, with periodic influx of cold polar waters of the Arctic Ocean.
Aasefjellet	79.52°N 10.70°E	36000	3	Both transects: Aasefjellet—west, 9 plots, descending to open sea.	Similar to those in Magdalenefjorden, but the area is directly exposed to the open sea.
Isfjorden	78.24°N 15.34°E	250	2	Platåberget—west, descending to sea inside the fjord, 9 plots; Platåberget—west, descending to valley bottom, 9 plots.	The warmest part of Svalbard due to the significant inflow of warm Atlantic water masses.
Hornsund	77.01°N 15.51°E	23500	3	Ariekammen—south-east, 9 plots; Fugleberget—south-east, 10 plots; Gnalberget—east, 7 plots. All descending to sea inside the fjord.	Influenced by the Sørkapp Current, carrying cold Arctic water masses from the north-western part of the Barents Sea to the north, with occasional inflows of warmer Atlantic waters from the West Spitsbergen Current.
Bjørnøya	74.38°N 19.03°E	10000	2	Both transects: Alfredfjellet—north, 5 plots descending to Lake Ellasjøen.	Situated close to the Atlantic water masses, but also partly influenced by cold Arctic waters; represents typical maritime climatic conditions.

*—(Keslinka et al. unpublished data)

### Data sampling

The study was conducted in July and August, during expeditions to Hornsund (2005, 2007), Magdalenefjorden and Aasefjelet (2007 and 2009), Bjørnøya (2008), and Isfjorden (2010). Samples were collected along 12 line transects established in the five locations described above. The upper parts of the transects consisted of vegetation-covered (to differing extents) rock debris, while the lower parts, approaching the sea/lake shore, were flat and more waterlogged. Each transect, depending on its local geomorphology condition, consisted of 5–10 plots (160×160 cm each) that were located from the respective transect’s starting point (plot 1) as follows: plot 2 (6 m), 3 (15 m), 4 (29 m), 5 (49 m), 6 (79 m), 7 (125 m), 8 (193 m), 9 (296 m), 10 (449 m), 11 (680 m), and 12 (1026 m), as described by Zwolicki et al. [15]. Within each sampling plot we assessed: (i) vegetation percentage cover, and collected (ii) three soil cores from three sites along the same diagonal of each plot (from the centre and from the two corners of each square), for physical, chemical and isotopic analyses, and (iii) three samples of taxa representing monocots, eudicots, mosses and lichens, for isotopic analyses.

### Guano deposition measurements

Guano deposition measurements were obtained in summer 2005 in Hornsund location only, during the study which compared the influence of guano deposition on soil chemistry between plankton-eater and fish-eater seabird colonies [15]. In the present study we used these data in order to reveal the relation between guano deposition rate and values of *δ*^15^N in the soil.

Guano deposition was assessed using black plastic sheets (150×150 cm) placed next to each sampling plot, along the colony and control transects. Exposure time depended on weather conditions, with the range of 20–36 hours. After exposure, a photograph of each sheet was taken and the sheets were cleaned for re-exposure. The area covered with birds’ droppings in each photograph was measured using SigmaScan Pro 5.0.0 software.

In order to provide repeatable estimates of guano deposition (dry mass) from the photographs, we performed an initial calibration by exposing stiff plastic sheets (150×150 cm), covered with very thin plastic film of a known mass. After these sheets were exposed and photographed, the plastic films were collected, dried and re-weighed to obtain the dry mass of droppings and to calculate a regression equation (*y* = 0.003 *x*, *R*^2^ = 0.7, n = 31; where: *x*—area covered by guano (cm^2^), and *y*—guano dry mass (g)) [15].

### Vegetation abundance and species composition

Within each sampling plot (total *n* = 97) we identified vascular plant species, and visually estimated the percentage contributions of individual species, and also the contributions of vascular plant and moss cover to the total vegetation cover. In order to describe changes in species α-diversity along the ornithogenically-influenced gradients, we decided to calculate Hill’s N2 diversity index, which measures the effective number of species and is linearly related to the number of species [29], separately for each plot (S1 File).

### Physicochemical soil properties

Each sample was collected with a shovel from the soil surface layer (to a depth of ca. 5–10 cm) and contained about 500 cm^3^ of soil. At sampling sites with very compact vegetation, we removed and discarded the upper layer of live and dead, poorly decomposed, plant material. Immediately after collection of samples (maximum 24 hours), in the field laboratory or in University of Svalbard, we took two sub-samples, 80 cm^3^ each, from each sample and performed assessment of:

1. soil moisture (%)—Soil sub-samples were weighed with electronic scales (precision 0.1 g) before and after oven-drying (60°C) to a constant mass. Soil moisture was defined as: soil moisture = ((wet mass—dry mass) dry mass^–1^) * 100%.
2. soil conductivity (μS cm^-1^) and pH—Soil samples of 80 cm^3^ were mixed with 160 cm^3^ of distilled water. The mixture was shaken for ca. 20 min and then filtered through a sieve (0.5 mm mesh). Conductivity and pH were quantified in the filtrate using a pH/conductivity/salinity meter CPC-401 (Elmetron) (S2 File).

### Stable isotope analyses

To assess *δ*^15^N and *δ*^13^C signatures in soil the remaining part of each soil sample was sieved (0.25 mm mesh) to remove stones and large plant debris, and ground with a vibrating mill (LMW-S, Testchem) to a grain size of less than 0.03 mm.

In case of plant and lichen tissues, we collected three samples from the aboveground parts of common vascular plants, mosses and lichens from each sample plot (not less than 5 mg dry mass in each sample). Immediately after collection they were manually cleaned of contaminants such as guano, soil particles etc., dried at 40–60°C to a constant mass and ground with a vibrating mill.

Prior to isotopic analyses we removed inorganic carbon (by adding 1 mL HCl 1N per 100 mg of soil) and lipids (using 4 ml of cyclohexane per 50 mg of soil). After this, a small amount of each sub-sample (1–2 mg, weighed with a microbalance, precision 0.001 mg) was packed into a tin capsule.

Nitrogen and carbon isotope ratios were determined by a continuous flow mass spectrometer (Thermo Fisher, Delta V Advantage) coupled to an elemental analyser (Thermo Fisher, Flash EA 1112). All samples for stable isotopes have been analyzed in one laboratory at the University of La Rochelle (France). Results were expressed in the conventional *δ*^15^N and *δ*^13^C notation, according to the equation: *δ* X = (*R*_*sample*_ *R*_*standard*_^-1^–1) 1,000 (‰), where *R*_*sample*_ was the stable isotope ratio ^15^N/^14^N or ^13^C/^12^C in the analysed sample, and *R*_*standard*_ was the stable isotope ratio ^15^N/^14^N or ^13^C/^12^C (respectively) in the reference material i.e. atmospheric N_2_ for nitrogen and PeeDee belemnite for carbon [23].

Within all 97 sample plots from five locations, we collected 1701 soil and plant samples for stable isotope analysis. Total nitrogen and carbon content (%) was also measured in each soil and vegetation sample during the isotopic analyses (S3 File).

### Statistical analyses and data management

In order to test the relationships between guano deposition and soil N and C contents and isotopic signatures, the non-parametric Spearman’s rank correlation was used (due to non-normal distributions of data and a relatively low number of sampling plots per group tested). This analysis was performed only in Hornsund location to validate that the value of *δ*^15^N was a good predictor of the ornithogenic fertilization (S4 File).

To explore individual soil and vegetation parameters and species response to soil *δ*^15^N we employed General Linear Models (GLM) to illustrate simple linear relation or Generalized Additive Models (GAM) if they better described species response curves than GLM. To find the best fit, Akaike Information Criterion (AIC) was performed, using Canoco 5.03 [30]. Models with scatter plots are available in supplementary materials (S1 Fig).

To explore the influence of theoretical environmental gradients in the data, and for comparison with constrained models (with the variability explained by specific environmental factors), unconstrained models were used (PCA—Principal Component Analysis—for N and C contents, and isotopic signatures of vascular plant, moss and lichen tissues; DCA—Detrended Correspondence Analysis—for plant community composition).

Depending on the length of the gradient obtained in DCA, measured in standard deviation (SD) units, Canonical Correspondence Analysis (CCA; when SD >3) or Redundancy Analysis (RDA; SD <3) were used to examine the influence of soil variables and the geographic location factor on vegetation properties [30]. After CCA or RDA, a Monte Carlo test with 499 permutations was performed to identify which of the factors significantly influenced the model. The efficiency of the environmental factor(s) in explaining the non-random variability existing in the data (%) was calculated by dividing the percentage variability explained by a given environmental factor by that explained by the first four axes in a PCA or DCA [30].

To calculate the unique contribution of seabird-derived matter in explaining the variability of vascular plant community composition as distinct from the effects of geographic location we used Variation Partitioning [31] based on two unimodal models: (1) with the four soil properties found to be significant in the previous constrained model, i.e. soil *δ*^15^N, moisture and pH, and plot order, and (2) with the five geographical locations. Four degrees of freedom (df) were used in each group to stabilize the model when the Monte Carlo permutation test was performed. For multiple comparisons we used Holm’s correction to control the family-wise type I errors [32].

To explore significant positive and negative relationships between individual plant species and soil *δ*^15^N values, a *t*-value biplot (with Van Dobben circles), which approximates the *t*-values of the regression coefficients of a weighted multiple regression, was generated [31].

The results were processed using the STATISTICA 9.1 package for correlations [33], and the CANOCO 5.03 package for ordination methods and regression models [30].

## Results

### Guano deposition, soil N and C contents and isotopic signatures

With increasing deposition of guano in Hornsund, the percentage of total soil nitrogen and carbon increased significantly (*r*_*s*_ = 0.70, *p* < 0.001; *r*_*s*_ = 0.71, *p* < 0.001; respectively). In this location the positive correlation between the deposition of bird faeces and soil nitrogen stable isotope ratio (*δ*^15^N) was also highly significant (*r*_*s*_ = 0.69, *n* = 20, *p* < 0.001), while it was negative for the carbon signature (*δ*^13^C; *r*_*s*_ = –0.70, *n* = 20, *p* < 0.001).

**Fig 2:**
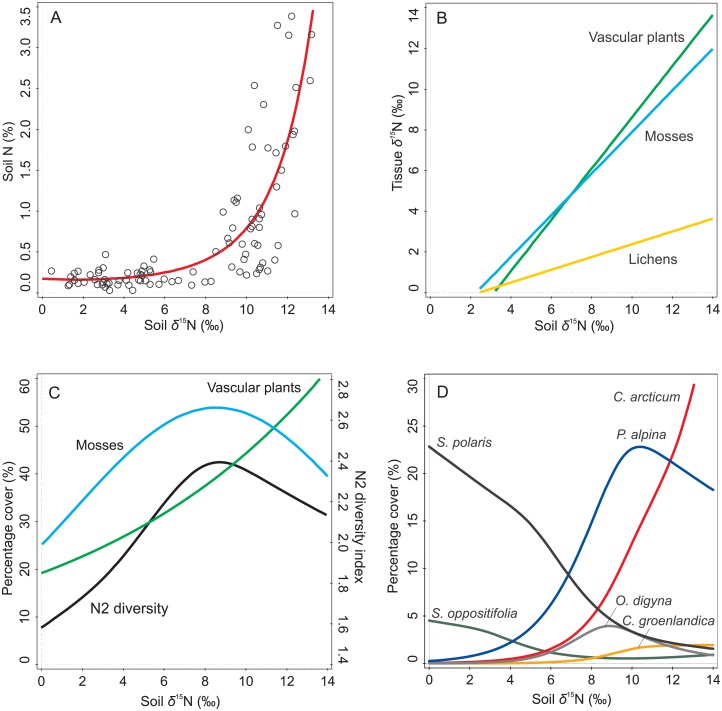
GAM and GLM response curves for increasing soil *δ*^15^N values: (A) total soil N content, (B) *δ*^15^N of vascular plants, mosses and lichens, (C) total percentage cover of vascular plants and mosses, and Hill’s N2 diversity index, (D) percentage cover of selected plant species (detailed results of the models are given in Table 2, B–D models with scatter plots are presented in the supplementary materials S1 Fig).

In all five locations studied, total soil N significantly increased with the higher soil *δ*^15^N values, starting to grow rapidly from 8‰ onwards (GAM, *p* < 0.001, Table 2, Fig 2A).

**Table 2:** GLM and GAM models results of the responses of the tested variables to soil *δ*^15^N (response curves given in Fig 2).

Response variable	Model	*R*^*2*^ (%)	*F*	*p*
Total soil N	GLM	71.5	116.7	<0.001
*δ*^15^N, Vascular plants	GLM	78.5	204.0	<0.001
*δ*^15^N, Mosses	GLM	51.3	59.0	<0.001
*δ*^15^N, Lichens	GLM	6.2	3.7	0.059
Vascular plant cover	GAM	18.9	24.0	<0.001
Moss cover	GAM	6.4	3.5	0.031
Hill N2 index	GAM	13	4.3	0.007
*Cerastium arcticum*	GAM	48.2	33.6	<0.001
*Cochlearia groenlandica*	GAM	30.7	14.3	<0.001
*Oxyria digyna*	GAM	30.9	17.5	<0.001
*Poa alpina*	GAM	48.4	47.9	<0.001
*Salix polaris*	GAM	29.4	13.1	<0.001
*Saxiphraga oppositifolia*	GAM	21.1	4.6	0.012

### Relationships between soil *δ*^15^N and vascular plant, moss and lichen N and C contents and isotopic signatures

**Fig 3:**
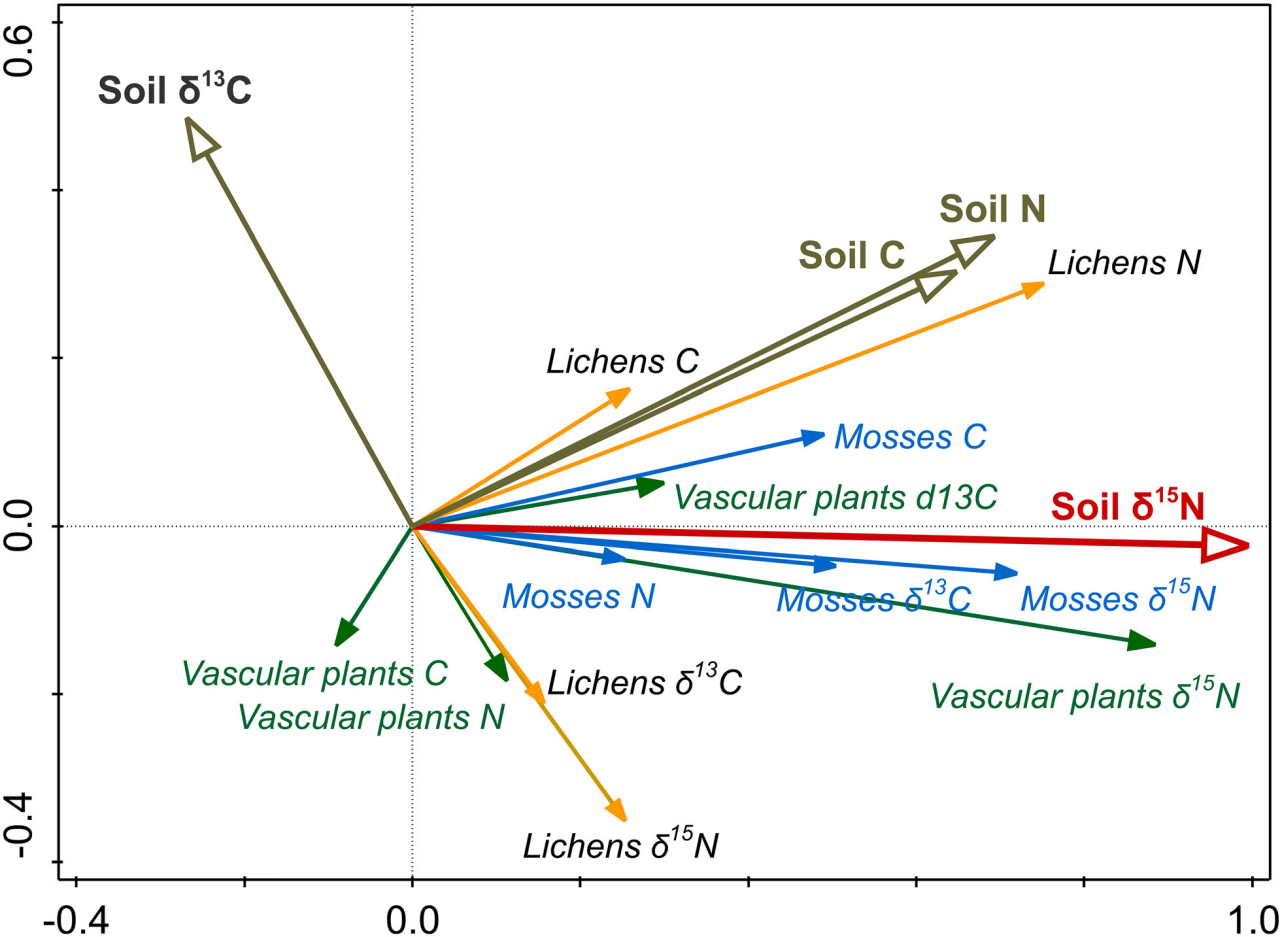
RDA ordination diagram based on N and C contents (%) and isotopic signatures (*δ*^15^N and *δ*^13^C, ‰) of vascular plants, mosses and lichens (response variables) in relation to the same parameters in soil (explanatory variables).

Soil nitrogen isotope value was linearly related to *δ*^15^N of vascular plants and mosses (GLM, *p* < 0.001), with these two groups showing almost identical trends. In the case of lichens, the relationship was not statistically significant (GLM, *p* = 0.059; Fig 2B, Table 2). Redundancy analysis (RDA) revealed that soil *δ*^15^N was the only factor contributing significantly to explaining the variability of total N and C contents and isotopic signatures of vascular plants, mosses and lichens, and accounted for 30.8% of the total variation of these parameters (Fig 3, Table 3).

**Table 3:** Total and explainable conditional effects of the environmental variables on vegetation total N and C content, *δ*^15^N and *δ*^13^C (RDA), and on vascular plant community composition (CCA).

			Variation (%)			
Response data	Model	Explanatory variable	Total	Efficiency^1^	*pseudo-F*	*p* (adj)^2^
Vascular plant, moss, and lichen N and C content, *δ*^15^N and *δ*^13^C	RDA					
		Soil *δ*^15^N	22.2	30.8	16	0.008
		Soil C content	2.2	3.1	1.6	0.342
		Soil *δ*^13^C	1.9	2.6	1.3	0.342
		Soil N content	1.5	2.1	1.1	0.342
Vascular plant community composition	CCA					
		Soil *δ*^15^N	10.3	34.1	10.9	<0.001
		Soil moisture	5.5	18.2	6.2	<0.001
		Plot order	2.8	9.3	3.2	<0.001
		pH	2.0	6.6	2.4	0.021
		Soil C content	1.7	5.6	2	0.066
		Conductivity	1.3	4.3	1.5	0.106
		Soil *δ*^13^C	0.7	2.3	0.9	0.486
		Soil N content	1.3	4.3	1.5	0.135

^1^ –the result of comparison with unconstrained model,

^2^ –adjusted by Holm correction.

### Responses of plant communities and individual species to soil *δ*^15^N values

Soil *δ*^15^N content was significantly and almost linearly related with the cover of vascular plants, and unimodally with the cover of mosses (GAM, *p* < 0.001 and *p* = 0.031, respectively; Fig 2C, Table 2). A similar unimodal, significant relationship was found between soil *δ*^15^N content and the Hill’s diversity coefficient (N2) (GAM, *p* = 0.013, Fig 2A, Table 2).

**Fig 4:**
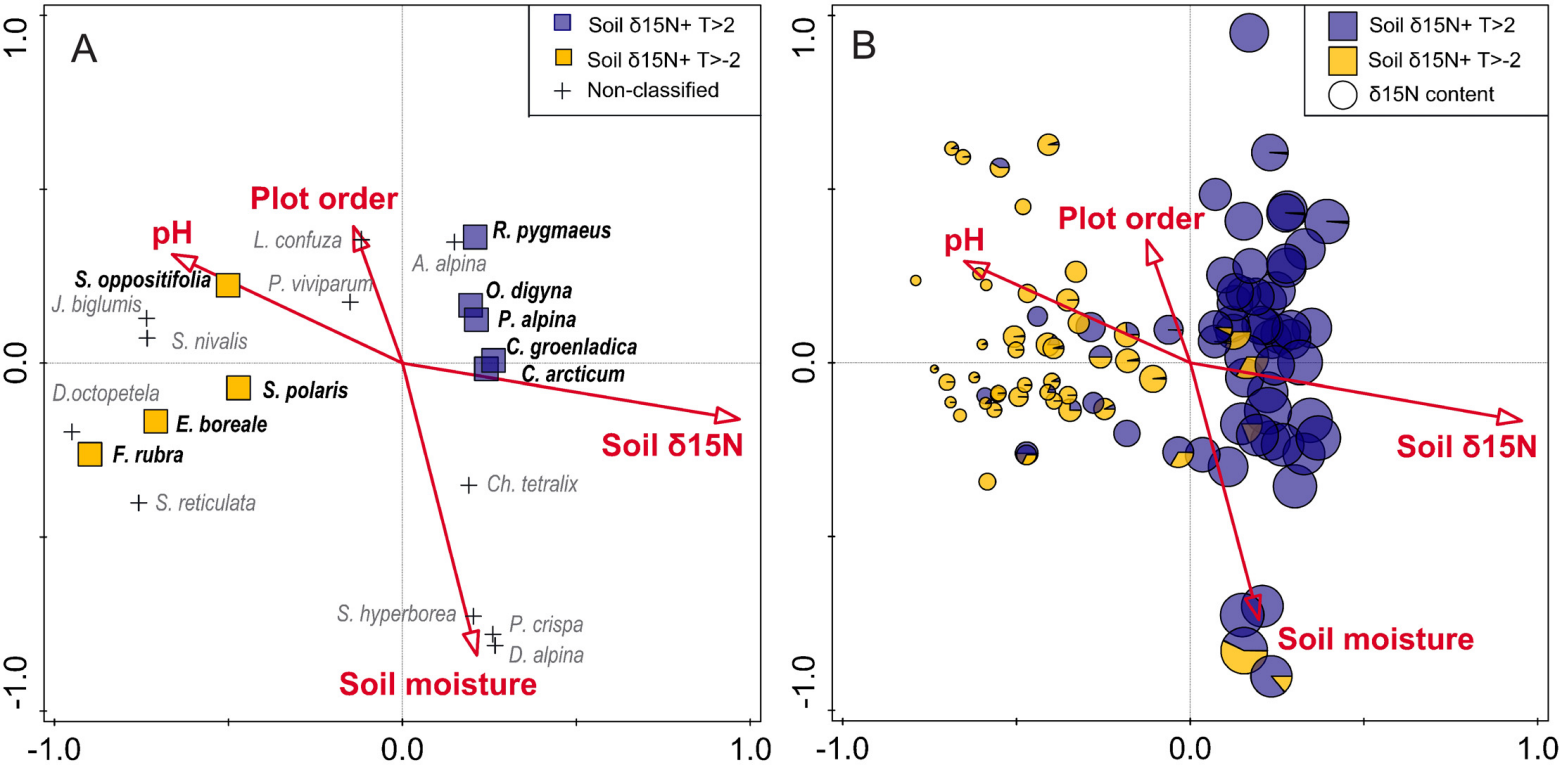
CCA ordination diagrams based on vascular plant community composition: (A) plant species centroids, with those related significantly positively (blue square) and negatively (yellow square) to soil *δ*^15^N (on the basis of *t*-value biplot); (B) sample plots with pie charts describing the total proportion of positively (blue) or negatively (yellow) related species to soil *δ*^15^N in plots. Circle size reflect soil *δ*^15^N content and red arrows indicate significant environmental variables.

The first four axes of DCA explained 30.9% of the total variability in vascular plant community composition. Of the eight tested soil properties, four significantly contributed to this variability (CCA; Table 3, Fig 4). The most important factor impacting community composition was soil *δ*^15^N, which explained 34.1% of the variability that was possible to explain (efficiency), followed by soil moisture (18.2%), plot order (9.3%), and pH (6.6%). The other four tested variables (total soil nitrogen and carbon, soil *δ*^13^C, conductivity) did not significantly influence community composition.

Based on the *t*-value biplot analysis performed for all 36 taxa of vascular plants, nine species were significantly correlated with soil *δ*^15^N (Fig 4). Five of them (*Ranunculus pygmaeus*, *Oxyria digyna*, *Poa alpina*, *Cerastium arcticum*, *Cochlearia groenlandica*) responded positively with increases in their percent cover. Negative relationships were found for four species (*Saxifraga oppositifolia*, *Festuca rubra*, *Salix polaris* and *Equisetum boreale*). These responses were particularly clear in the common species, *C*. *arcticum*, *P*. *alpina* and *S*. *polaris* (Figs 2D and 4A, Table 2).

### The relative importance of soil properties and geographical location for plant community composition

**Fig 5:**
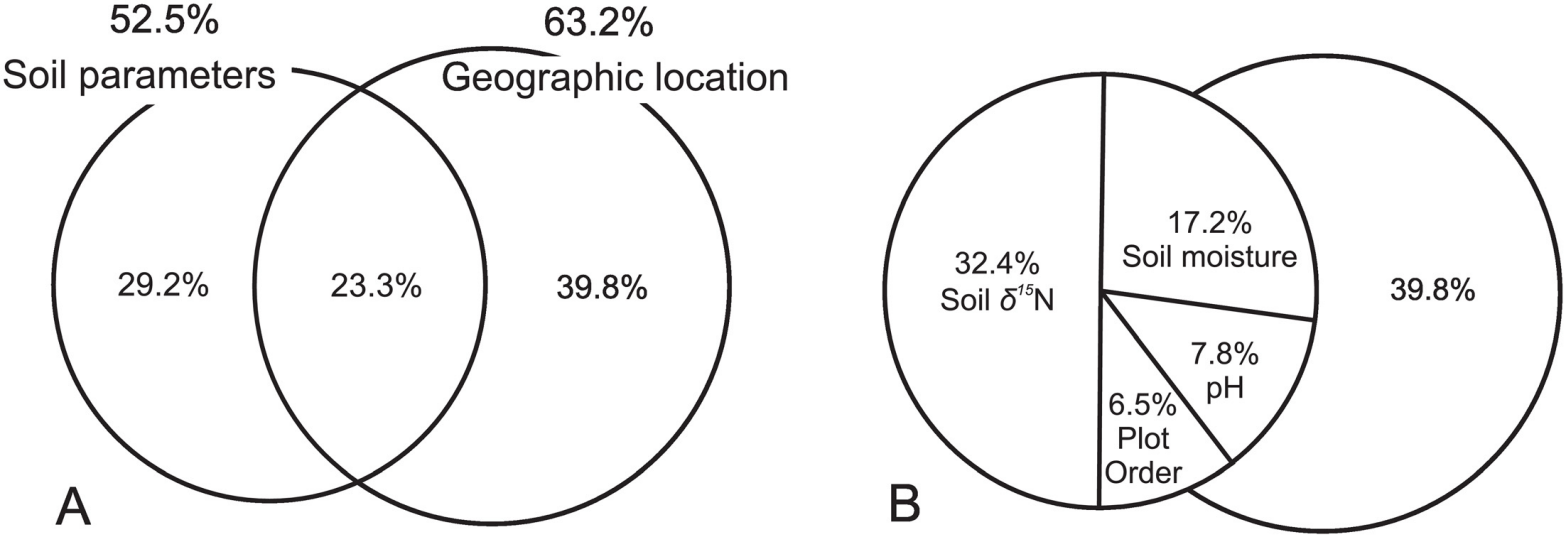
Unique and shared fractions of the total variation of vascular plant community composition, explained by geographic location and soil parameters (*δ*^15^N, moisture, pH and plot order) (A), with the contribution of each soil parameter to the variation in the soil properties (B).

Four statistically important factors influencing vascular plant community composition, i.e. *δ*^15^N content, soil moisture, pH and plot order (Table 3), and five geographical locations, explained in total 92.3% of the variability of the plant community. The variation partitioning test (*p* < 0.01) revealed that the soil properties explained 52.5%, while the location factor accounted for 63.2%, of this variability. The two groups of factors shared 23.3% of the explained variation (Fig 5A). Soil *δ*^15^N was responsible for 32.4%, moisture accounted for 17.2%, pH for 7.8% and plot order for 6.5% of the variation contributed by soil properties (Fig 5B).

## Discussion

One of the most important general features of polar terrestrial ecosystems is their chronically low nutrient availability to plants, through the poor quality of the soil which also spends much of the year frozen [34]. Therefore, any additional nutrient sources such as seabird guano are of great importance for ecosystem functioning [18,28,35]. This study presents direct evidence of a strong relation between the proportion of nitrogen stable isotopes in soil and the level of seabird guano deposition, and confirms previous studies proposing that *δ*^15^N signature is a reliable measure of maritime nitrogen supply by seabirds [36–40].

The highest value of soil *δ*^15^N found in our study was 13.7‰, similar to that reported by Skrzypek et al. 2015 [40]. The value is considerably higher than that of the little auk faeces itself reported by those authors (8.1‰), probably as a consequence of microbial activity in the soil followed by ammonia volatilization [41]. Soil *δ*^15^N values exceeding 8‰ could also be influenced by other nitrogen sources present in the area, such as little auk carcasses, in which the N isotopic ratio in muscle tissue may reach 11.2‰ [42], and in feathers 11.4‰ [43].

The *δ*^15^N ratio was strongly correlated with total nitrogen amount in the soil, which confirms a general assumption that seabirds as a biovectors are locally the most important source of nutrients in Arctic tundra ecosystems [15]. Furthermore, the levels of *δ*^15^N in the tissues of vascular plants and mosses were linearly related with *δ*^15^N of the underlying soil. Finally, nitrogen isotopes in soil was the most important variable explained by the total amounts of N and their stable isotope signatures in vegetation. These relationships indicate that plants easily incorporate and rely on this source of nitrogen [44,45]. The slight differences between the relationships shown by vascular plants and mosses may be connected with the different N-loading strategies used by the two groups, i.e. absorption through the roots by vascular plants, and via the surface of leaves by mosses [46]. The relation was insignificant for lichens probably due to the utilization of atmospheric nitrogen fixation [47].

Our results indicate that the carbon stable isotope is not the best indicator of the influence of seabirds. We have not found any significant relations between *δ*^13^C values in the soil and in the plant tissues. This was also an insignificant variable in explaining plant community composition. Primarily, we expected that the content of ^13^C would be greater in the matter originating from the sea [26,48]. However, many authors faced similar difficulties with interpretation of carbon isotope results (e.g. [48–50]).

The data obtained in this study demonstrate that tundra communities vary mostly between different geographical locations. However, at a local scale we documented that nutrient input, which little auks were primarily responsible for, created the most important gradients of all the tested variables, and explained the largest proportion of variation in the plant community. Our variation partitioning model characterized almost maximal efficiency (92.3%) which implies that it includes most of the environmental factors that influence the variability of studied tundra vegetation. The fraction shared between geographical location and soil variables probably results from the differences in colony size and fertilization level between locations.

Besides having isotopically enriched tissues, vascular plants and mosses clearly responded to the ornithogenic nitrogen input through increases in abundance. Total cover of vascular plants increased linearly with soil *δ*^15^N by up to 60%, when the nitrogen isotopic ratio achieved its maximum (almost 14‰). Total moss percentage cover also increased with soil *δ*^15^N level but reached the highest values (ca. 55%) around *δ*^15^N = 8‰, then began to decrease. This domination of vascular plants in highly fertilized conditions could be the result of their much faster nitrogen uptake allowing them to outcompete mosses [51]. The peak of the plant diversity (Hill’s index) for the community was also very close to *δ*^15^N = 8‰. It is very suggestive that this tipping point is associated with the *δ*^15^N signature of bird faeces, determined in a previous study to be 8.1±0.5‰ in the Hornsund area [40].

This critical N isotopic level appeared to have a strong influence on the proportions of particular plant species in the community. Along the *δ*^15^N gradient in the soil, plant species distributions demonstrate clear niche segregation. Changes in nutrient availability should impact on interspecific competition, as species with an ability to respond rapidly to increased nutrients will have a competitive advantage over those adapted to low nutrient levels [51,52]. This should result in selection for plants with high growth rates and rapid tissue turnover but low nutrient use efficiencies (e.g. *C*. *groenlandica*, *P*. *alpina*, *C*. *arcticum*, and *O*. *digyna* in this study), and against slow-growing plants with long-lived leaves and high nutrient use efficiency (*S*. *oppositifolia* and *S*. *polaris* here) [53,54]. Field observations and manipulation experiments in the Arctic Aleutian Islands showed that some graminoid species spread rapidly in response to increasing amounts of seabird-derived nutrients, outcompeting slower-growing dwarf shrubs and forbs, and forming lush stands of grasses and sedges with reduced overall plant diversity [55].

The responses of nine vascular plant species in the current study (25% of the vascular plant taxa recorded) were significantly related to ornithogenic fertilization. *S*. *polaris* and *S*. *oppositifolia* are typical of species growing in poor nutrient conditions, with their highest percentage covers observed under the lowest *δ*^15^N values (from 2 to 4‰). These perennial dwarf woody shrubs are widespread in Arctic deserts, and are well adapted to unpredictable resource availability and limitation of growth [53]. They exemplify stress-tolerant plants which are able to store their restricted resources [56]. With increasing nutrient input we observed that the coverage of these plants declined, and there was a rapid increase and domination of other species, especially *P*. *alpina* (at levels from 6 to 10‰) and *C*. *arcticum* (from 8 to 13‰). *P*. *alpina* was first described as a dominant species in nitrophilous communities by Summerhayes and Elton [57], and *C*. *arcticum* is also known as a very abundant species occurring in the vicinity of bird colonies [58,59].

Many of the vascular plants that typify ornithogenic tundra [59] are reported to be important food for large herbivores (reindeer, geese, ptarmigan, on Svalbard). These fast-growing, nutritious and palatable plant species create large grazing areas of good quality in the vicinity of seabird colonies [60,61]. Previous studies have also shown that areas without seabirds are characterized by low-productivity tundra dominated by dwarf shrubs and forbs [18,55,62]. Similarly, on sub-Antarctic islands, nutrient subsidies from seabirds encourage a change from carpets of ferns and mosses to lush tussock grasslands [37,63]. Such selection for fast-growing graminoids in response to high nitrogen availability is typical for cold regions where annual productivity is low [26,64]. In our study, beside the grass species, the ornithogenic N-input favours species such as *C*. *groenlandica* and *O*. *digyna*, that probably do not rely exclusively on fungal or bacterial symbionts for nutrient acquisition [65,66]. As with the graminoids, both these species are characterized by high nutrient contents and relative growth rates, as well as rapid maturation and high fecundity [14].

Nutrient enrichment derived from colonial seabirds typically enhances above-ground biomass, while also locally reducing plant diversity if fertilization is considerable [27,67]. We observed this phenomenon in the areas of the highest soil *δ*^15^N, where *α* diversity was lowered. In a large scale seabirds may increase *β* or *γ* plants diversity by favouring the occurrence and enhancing the abundance of some nitrophilous plant species that are relatively widespread, although locally in areas that are not ornithogenically-influenced can have very low abundance on like. *O*. *digyna*, *C*. *groenladica* or *C*. *arcticum*.

The present study is of particular importance given the recent strong climatic and oceanographic changes in the Arctic which influence both marine and terrestrial parts of the ecosystem. Forecasts for zooplankton communities suggests these changes will lead to decreasing availability of *Calanus glacialis*—the staple food of little auks, and consequently a decline of the auk population [21,68,69]. This may lead to reduction in ornithogenic nutrient transfer onto land, impacting the tundra plant and animal communities [70,71]

## Supporting Information

S1 Fig GLM and GAM models from Fig 3B–3D with scatter plots. (TIF)

S1 File Dataset table of plant community composition based on percentage ground cover (response variables). (XLSX)

S2 File Dataset table of environmental data (explanatory variables). (XLSX)

S3 File Dataset table of N and C contents (%) and isotopic signatures (δ15N and δ13C, ‰) of vascular plants, mosses and lichens tissues (response variables). (XLSX)

S4 File Dataset table of guano deposition in Hornsund location and C and N elements and their stable isotopes in soil. (XLSX)

## References

[1] Odum E. Ecology and our endangered life-support systems. Sunderland, Mass.: Sinauer Associates; 1989.

[2] Rannie W. Summer Air Temperature and Number of Vascular Species in Arctic Canada. Arctic. 1986;39(2).

[3] Walker M. Patterns and causes of Arctic plant community diversity In: Chapin F, Kӧrner C, ed. by. Arctic and alpine biodiversity: Pattern, causes and ecosystem consequences. 1st ed Berlin: SpringerVerlag; 2015 p. 320.

[4] Raup H. The relation of the vascular flora to some factors of site in the Mesters Vig district, Northeast Greenland. København: C.A. Reitzel; 1969.

[5] Gould W, Walker M. Plant communities and landscape diversity along a Canadian Arctic river. Journal of Vegetation Science. 1999;10(4):537–548.

[6] Kӧrner C. Alpine plant life. Berlin: Springer; 2003.

[7] Wahren C, Walker M, Bret-Harte M. Vegetation responses in Alaskan arctic tundra after 8 years of a summer warming and winter snow manipulation experiment. Global Change Biol. 2005;11(4):537–552.

[8] Frenot Y, Gloaguen J, Cannavacciuolo M, Bellido A. Primary succession on glacier forelands in the subantarctic Kerguelen Islands. Journal of Vegetation Science. 1998;9(1):75–84.

[9] Hoffmann M. Not across the North Pole: Plant migration in the Arctic. New Phytologist. 2011;193(2):474–480. 10.1111/j.1469-8137.2011.03924.x 21988606

[10] Cannone N, Guglielmin M, Gerdol R. Relationships between vegetation patterns and periglacial landforms in northwestern Svalbard. Polar Biol. 2004;27(9).

[11] Broady P. Broadscale patterns in the distribution of aquatic and terrestrial vegetation at three ice-free regions on Ross Island, Antarctica. Hydrobiologia. 1989;172(1):77–95.

[12] Kennedy A. Water as a Limiting Factor in the Antarctic Terrestrial Environment: A Biogeographical Synthesis. Arctic and Alpine Research. 1993;25(4):308.

[13] Wait D, Aubrey D, Anderson W. Seabird guano influences on desert islands: soil chemistry and herbaceous species richness and productivity. Journal of Arid Environments. 2005;60(4):681–695.

[14] Ellis J, Bellingham P, Cameron D, Croll D, Kolb G, Kueffer C, et al Effects of seabirds on plant communities In: Mulder C, Anderson W, Towns D, Bellingham P, ed. by. Seabird islands. Ecology, invasion and restoration. New York: Oxford University Press; 2011 p. 135–176.

[15] Zwolicki A, Zmudczyńska-Skarbek K, Iliszko L, Stempniewicz L. Guano deposition and nutrient enrichment in the vicinity of planktivorous and piscivorous seabird colonies in Spitsbergen. Polar Biol. 2013;36(3):363–372.

[16] Zwolicki A, Barcikowski M, Barcikowski A, Cymerski M, Stempniewicz L, Convey P. Seabird colony effects on soil properties and vegetation zonation patterns on King George Island, Maritime Antarctic. Polar Biol. 2015;38(10):1645–1655.

[17] Wojciechowska A, Zwolicki A, Barcikowski A, Stempniewicz L. The structure of *Cochlearia groenlandica* population along the bird colony influence gradient (Hornsund, Spitsbergen). Polar Biol. 2015;38(11):1919–1930.

[18] Croll D. Introduced Predators Transform Subarctic Islands from Grassland to Tundra. Science. 2005;307(5717):1959–1961. 15790855 10.1126/science.1108485

[19] Zmudczyńska K, Olejniczak I, Zwolicki A, Iliszko L, Convey P, Stempniewicz L. Influence of allochtonous nutrients delivered by colonial seabirds on soil collembolan communities on Spitsbergen. Polar Biol. 2012;35(8):1233–1245.

[20] Wojczulanis-Jakubas K, Jakubas D, Welcker J, Harding A, Karnovsky N, Kidawa D, et al Body size variation of a high-Arctic seabird: the dovekie (Alle alle). Polar Biol. 2011;34(6):847–854.

[21] Kwaśniewski S, Głuchowska M, Jakubas D, Wojczulanis-Jakubas K, Walkusz W, Karnovsky N, et al The impact of different hydrographic conditions and zooplankton communities on provisioning Little Auks along the West coast of Spitsbergen. Progress in Oceanography. 2010;87(1–4):72–82.

[22] Stempniewicz L. Biomass of Dovekie Excreta in the Vicinity of a Breeding Colony. Colonial Waterbirds. 1990;13(1):62.

[23] Kelly J. Stable isotopes of carbon and nitrogen in the study of avian and mammalian trophic ecology. Can J Zool. 2000;78(1):1–27.

[24] Dawson T, Mambelli S, Plamboeck A, Templer P, Tu K. Stable Isotopes in Plant Ecology. Annu Rev Ecol Syst. 2002;33(1):507–559.

[25] Harding J, Hawke D, Holdaway R, Winterbourn M. Incorporation of marine-derived nutrients from petrel breeding colonies into stream food webs. Freshwater Biology. 2004;49(5):576–586.

[26] Huiskes A, Boschker H, Lud D, Moerdijk-Poortvliet T. Stable isotope ratios as a tool for assessing changes in carbon and nutrient sources in Antarctic terrestrial ecosystems. Plant Ecol. 2006.

[27] Ellis J, Farina J, Witman J. Nutrient transfer from sea to land: the case of gulls and cormorants in the Gulf of Maine. Journal of Animal Ecology. 2006;75(2):565–574. 16638009 10.1111/j.1365-2656.2006.01077.x

[28] Zmudczyńska-Skarbek K, Balazy P, Kuklinski P. An assessment of seabird influence on Arctic coastal benthic communities. Journal of Marine Systems. 2015;144:48–56.

[29] Hill M. Diversity and Evenness: A Unifying Notation and Its Consequences. Ecology. 1973;54(2):427.

[30] Ter Braak C, Smilauer P. Canoco reference manual and user’s guide: software for ordination. Ithaca, NY, USA: Microcomputer Power; 2012.

[31] Legendre P. Studying beta diversity: ecological variation partitioning by multiple regression and canonical analysis. Journal of Plant Ecology. 2007;1(1):3–8.

[32] Holm S. A simple sequentially rejective multiple test procedure. Scandinavian journal of statistics. 1979;65–70.

[33] STATISTICA (data analysis software system), version 9.1. 2010.

[34] Thomas D, Fogg G. The biology of polar regions. Oxford: Oxford University Press; 2008.

[35] Ryan P, Watkins B. The influence of physical factors and ornithogenic products on plant and arthropod abundance at an Inland Nunatak group in Antarctica. Polar Biol. 1989;10(2).

[36] Bokhorst S, Huiskes A, Convey P, Aerts R. External nutrient inputs into terrestrial ecosystems of the Falkland Islands and the Maritime Antarctic region. Polar Biol. 2007;30(10):1315–1321.

[37] Erskine P, Bergstrom D, Schmidt S, Stewart G, Tweedie C, Shaw J. Subantarctic Macquarie Island—a model ecosystem for studying animal-derived nitrogen sources using 15 N natural abundance. Oecologia. 1998;117(1–2):187–193. 28308485 10.1007/s004420050647

[38] Cocks M, Balfour D, Stock W. On the uptake of ornithogenic products by plants on the inland mountains of Dronning Maud Land, Antarctica, using stable isotopes. Polar Biology. 1998;20(2):107–111.

[39] Kolb G, Young H, Anderson W. Effects of seabirds on island consumers In: Mulder C, Anderson W, Towns D, Bellingham P, ed. by. Seabird islands Ecology, invasion, and restoration. 1st ed New York: Oxford University Press; 2011 p. 212–241.

[40] Skrzypek G, Wojtuń B, Richter D, Jakubas D, Wojczulanis-Jakubas K, Samecka-Cymerman A. Diversification of Nitrogen Sources in Various Tundra Vegetation Types in the High Arctic. PLOS ONE. 2015;10(9):e0136536 10.1371/journal.pone.0136536 26376204 PMC4574312

[41] Ansari A, Hodson A, Heaton T, Kaiser J, Marca-Bell A. Stable isotopic evidence for nitrification and denitrification in a High Arctic glacial ecosystem. Biogeochemistry. 2013;113(1–3):341–357.

[42] Fort J, Cherel Y, Harding A, Welcker J, Jakubas D, Steen H, et al Geographic and seasonal variability in the isotopic niche of little auks. Marine Ecology Progress Series. 2010;414:293–302.

[43] Burke CM, Montevecchi WA, Hedd A, McFarlane-Tranquilla LA, Regular PM, Robertson GJ, et al Age-specific variation in trophic niche overlap of Dovekies Alle Alle. Marine Ornithology. 2014;42(1):17–22.

[44] Kolb G, Ekholm J, Hambäck P. Effects of seabird nesting colonies on algae and aquatic invertebrates in coastal waters. Marine Ecology Progress Series. 2010;417:287–300.

[45] Signa G, Mazzola A, Vizzini S. Effects of a small seagull colony on trophic status and primary production in a Mediterranean coastal system (Marinello ponds, Italy). Estuarine, Coastal and Shelf Science. 2012;111:27–34.

[46] Glime J. Bryophyte ecology, vol 1. Physiological ecology (Internet). 1st ed Ebook sponsored by Michigan Technological University and the International Association of Bryologists; 2007 Available: http://www.bryoecol.mtu.edu.

[47] Nash T. Lichen biology. Cambridge: Cambridge University Press; 1996.

[48] Harding J, Hawke D, Holdaway R, Winterbourn M. Incorporation of marine-derived nutrients from petrel breeding colonies into stream food webs. Freshwater Biology. 2004;49(5):576–586.

[49] Cocks M, Balfour D, Stock W. On the uptake of ornithogenic products by plants on the inland mountains of Dronning Maud Land, Antarctica, using stable isotopes. Polar Biology. 1998;20(2):107–111.

[50] Hawke D, Newman J. Carbon‐13 and nitrogen‐15 enrichment in coastal forest foliage from nutrient‐poor and seabird‐enriched sites in southern New Zealand. New Zealand Journal of Botany. 2007;45(2):309–315.

[51] Hill P, Farrar J, Roberts P, Farrell M, Grant H, Newsham K, et al Vascular plant success in a warming Antarctic may be due to efficient nitrogen acquisition. Nature Climate Change. 2011;1(1):50–53.

[52] Tilman D. Resources, competition and the dynamics of plant communities In: Crawley M, ed. by. Plant Ecology. 1st ed Oxford: Blackwell Scientific Publications; 1986 p. 51–75.

[53] Grime J. Competition and the struggle for existence In: Andersson R, Turner B, Taylor L, ed. by. Population dynamics. 1st ed 1979 p. 123–139.

[54] Chapin F. The Mineral Nutrition of Wild Plants. Annu Rev Ecol Syst. 1980;11(1):233–260.

[55] Maron J, Crone E. Herbivory: effects on plant abundance, distribution and population growth. Proceedings of the Royal Society B: Biological Sciences. 2006;273(1601):2575–2584. 17002942 10.1098/rspb.2006.3587 PMC1635468

[56] Chapin F. The ecology and economics of storage in plants. Annual Review of Ecology and Systematics. 1990;21(1):423–447.

[57] Summerhayes V, Elton C. Further contributions to the ecology of S pitsbergen. Journal of Ecology. 1928;16:193–268.

[58] Odasz A. Nitrate reductase activity in vegetation below an arctic bird cliff, Svalbard, Norway. Journal of Vegetation Science. 1994;5(6):913–920. 10.2307/3236203 PMC7201890 32390712

[59] Eurola S, Hakala A. The bird cliff vegetation of Svalbard. Aquilo Seria Botanica. 1977;15, p.l–18.

[60] Bryant J, Chapin F, Klein D. Carbon/Nutrient Balance of Boreal Plants in Relation to Vertebrate Herbivory. Oikos. 1983;40(3):357.

[61] Staaland H, Brattbakk I, Ekern K, Kildemo K. Chemical composition of reindeer forage plants in Svalbard and Norway. Ecography. 1983;6(2):109–122.

[62] Zelenskaya L, Khoreva M. Growth of the nesting colony of slaty-backed gulls (Larus schistisagus) and plant cover degradation on Shelikan Island (Taui inlet, the Sea of Okhotsk). Russ J Ecol. 2006;37(2):126–134.

[63] Wace N. The Vegetation of Gough Island. Ecological Monographs. 1961;31(4):337.

[64] Wookey P, Aerts R, Bardgett R, Baptist F, Brathenn K, Cornelissen J, et al Ecosystem feedbacks and cascade processes: understanding their role in the responses of Arctic and alpine ecosystems to environmental change. Global Change Biology. 2009;15(5):1153–1172.

[65] Cazares E, Trappe J, Jumpponen A. Mycorrhiza-plant colonization patterns on a subalpine glacier forefront as a model system of primary succession. Mycorrhiza. 2005;15(6):405–416. 15772815 10.1007/s00572-004-0342-1

[66] Delaux P, Varala K, Edger P, Coruzzi G, Pires J, Ané J. Comparative Phylogenomics Uncovers the Impact of Symbiotic Associations on Host Genome Evolution. PLoS Genetics. 2014;10(7):e1004487 10.1371/journal.pgen.1004487 25032823 PMC4102449

[67] Gillham M. Alteration of the Breeding Habitat by Sea-Birds and Seals in Western Australia. The Journal of Ecology. 1961;49(2):289.

[68] Carstensen J, Weydmann A, Olszewska A, Kwasniewski S. Effects of environmental conditions on the biomass of Calanus spp. in the Nordic Seas. Journal of Plankton Research. 2012;34(11):951–966.

[69] Weydmann A, Carstensen J, Goszczko I, Dmoch K, Olszewska A, Kwasniewski S. Shift towards the dominance of boreal species in the Arctic: inter-annual and spatial zooplankton variability in the West Spitsbergen Current. Marine Ecology Progress Series. 2014;501:41–52.

[70] Stempniewicz L, Błachowiak-Samołyk K, Węsławski J. Impact of climate change on zooplankton communities, seabird populations and arctic terrestrial ecosystem-A scenario. Deep Sea Research Part II: Topical Studies in Oceanography. 2007;54(23–26):2934–2945.

[71] Jakubas D, Zmudczyńska K, Wojczulanis-Jakubas K, Stempniewicz L. Faeces deposition and numbers of vertebrate herbivores in the vicinity of planktivorous and piscivorous seabird colonies in Hornsund, Spitsbergen. Polish Polar Research. 2008;29:45–58.

